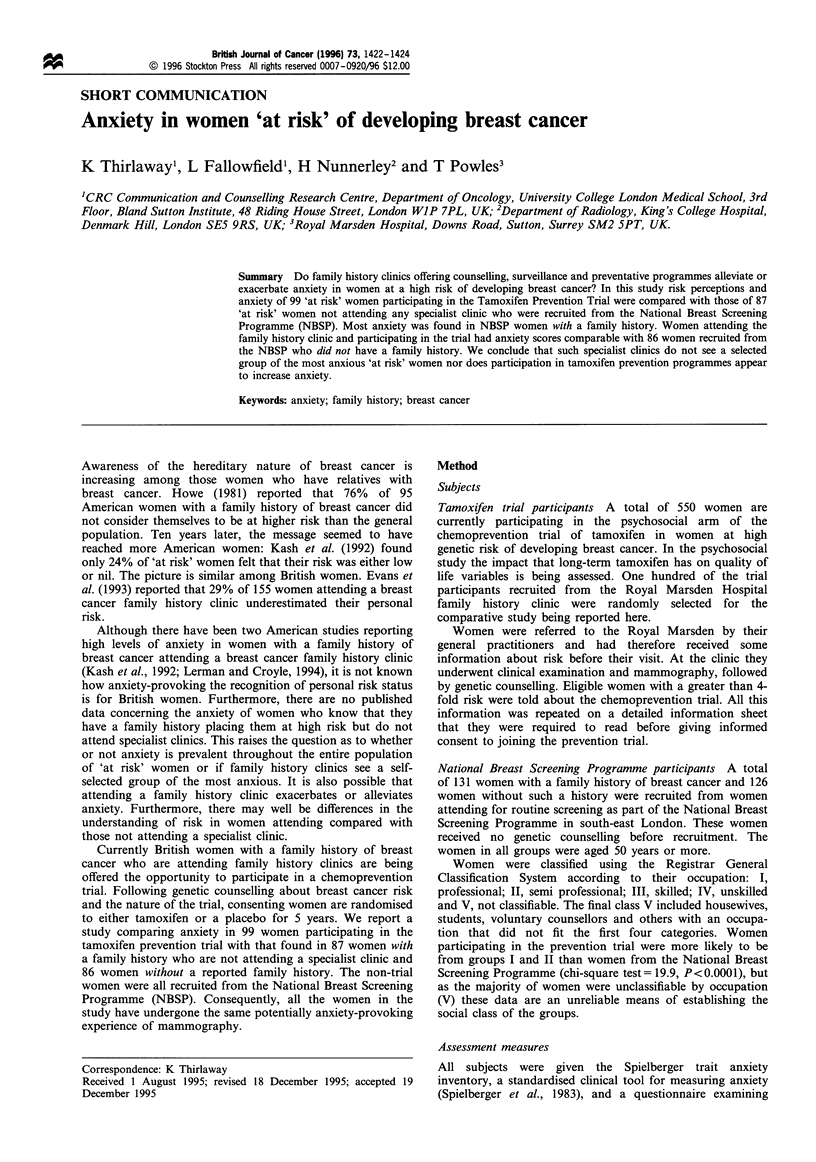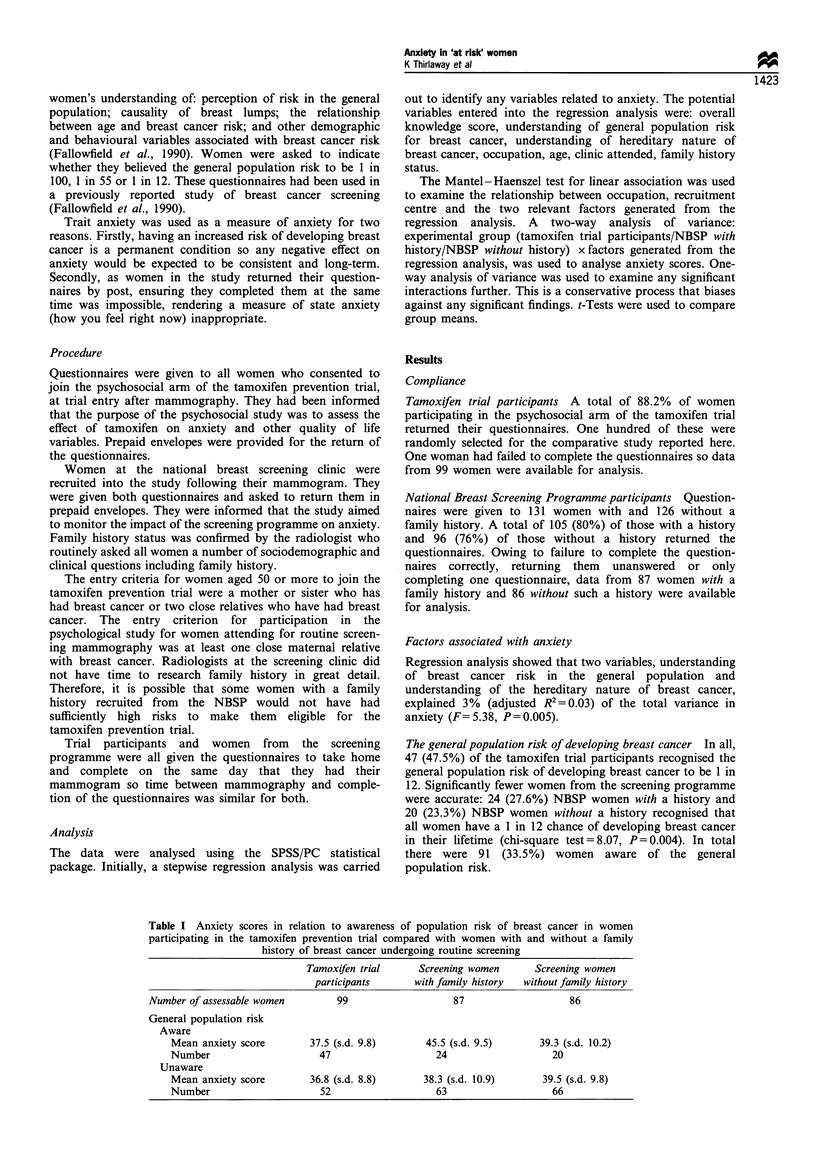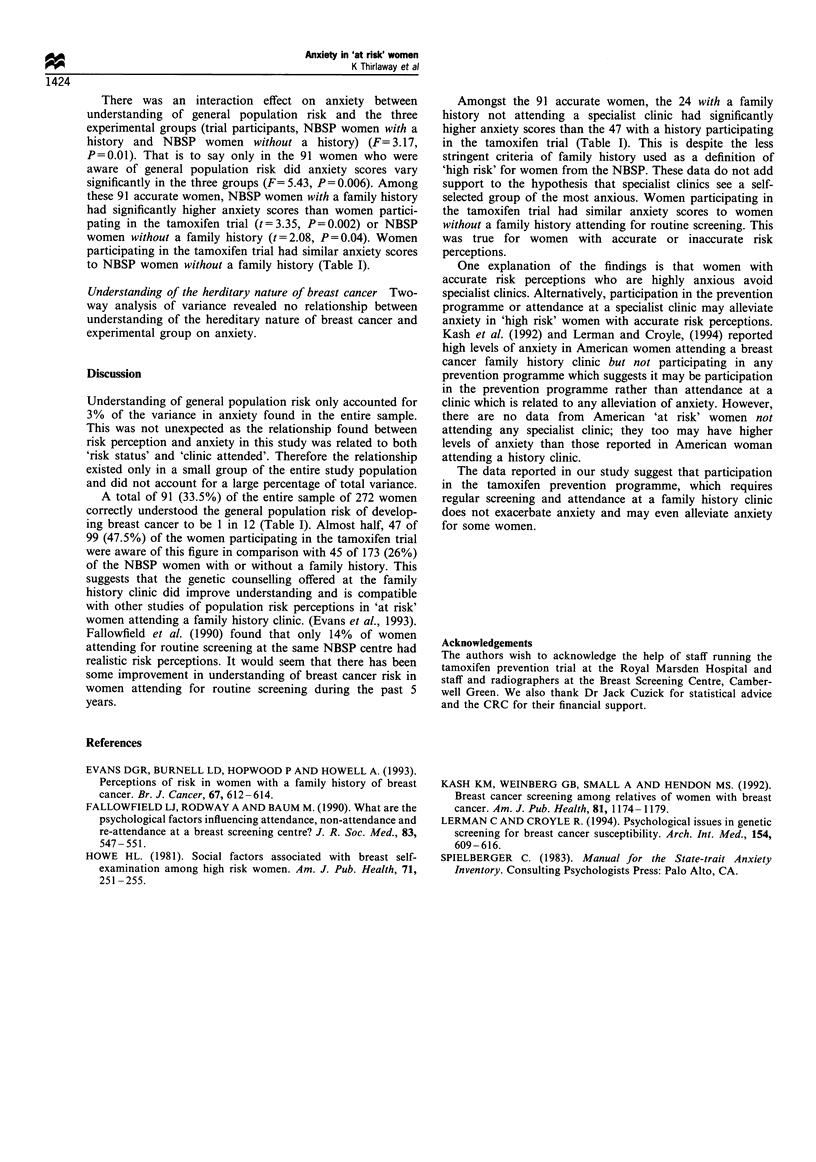# Anxiety in women "at risk' of developing breast cancer.

**DOI:** 10.1038/bjc.1996.269

**Published:** 1996-06

**Authors:** K. Thirlaway, L. Fallowfield, H. Nunnerley, T. Powles

**Affiliations:** Department of Oncology, University College London Medical School, UK.

## Abstract

Do family history clinics offering counselling, surveillance and preventative programmes alleviate or exacerbate anxiety in women at a high risk of developing breast cancer? In this study risk perceptions and anxiety of 99 'at risk' women participating in the Tamoxifen Prevention Trial were compared with those of 87 'at risk' women not attending any specialist clinic who were recruited from the National Breast Screening Programme (NBSP). Most anxiety was found in NBSP women with a family history. Women attending the family history clinic and participating in the trial had anxiety scores comparable with 86 women recruited from the NBSP who did not have a family history. We conclude that such specialist clinics do not see a selected group of the most anxious 'at risk' women nor does participation in tamoxifen prevention programmes appear to increase anxiety.


British Journal of Cancer (1996) 73, 1422-1424
© 1996 Stockton Press All rights reserved 0007-0920/96 $12.00

SHORT COMMUNICATION

### Anxiety in women 'at risk' of developing breast cancer

K Thirlaway[1], L Fallowfield[1], H Nunnerley[2] and T Powles[3]

[1]*CRC Communication and Counselling Research Centre, Department of Oncology, University College London Medical School, 3rd Floor, Bland Sutton Institute, 48 Riding House Street, London W1P 7PL, UK;* [2]*Department of Radiology, King's College Hospital, Denmark Hill, London SE5 9RS, UK;* [3]*Royal Marsden Hospital, Downs Road, Sutton, Surrey SM2 5PT, UK.*

**Summary** Do family history clinics offering counselling, surveillance and preventative programmes alleviate or exacerbate anxiety in women at a high risk of developing breast cancer? In this study risk perceptions and anxiety of 99 'at risk' women participating in the Tamoxifen Prevention Trial were compared with those of 87 'at risk' women not attending any specialist clinic who were recruited from the National Breast Screening Programme (NBSP). Most anxiety was found in NBSP women *with* a family history. Women attending the family history clinic and participating in the trial had anxiety scores comparable with 86 women recruited from the NBSP who *did not* have a family history. We conclude that such specialist clinics do not see a selected group of the most anxious 'at risk' women nor does participation in tamoxifen prevention programmes appear to increase anxiety.

**Keywords:** anxiety; family history; breast cancer

Awareness of the hereditary nature of breast cancer is increasing among those women who have relatives with breast cancer. Howe (1981) reported that 76% of 95 American women with a family history of breast cancer did not consider themselves to be at higher risk than the general population. Ten years later, the message seemed to have reached more American women: Kash *et al.* (1992) found only 24% of 'at risk' women felt that their risk was either low or nil. The picture is similar among British women. Evans *et al.* (1993) reported that 29% of 155 women attending a breast cancer family history clinic underestimated their personal risk.

Although there have been two American studies reporting high levels of anxiety in women with a family history of breast cancer attending a breast cancer family history clinic (Kash *et al.*, 1992; Lerman and Croyle, 1994), it is not known how anxiety-provoking the recognition of personal risk status is for British women. Furthermore, there are no published data concerning the anxiety of women who know that they have a family history placing them at high risk but do not attend specialist clinics. This raises the question as to whether or not anxiety is prevalent throughout the entire population of 'at risk' women or if family history clinics see a self-selected group of the most anxious. It is also possible that attending a family history clinic exacerbates or alleviates anxiety. Furthermore, there may well be differences in the understanding of risk in women attending compared with those not attending a specialist clinic.

Currently British women with a family history of breast cancer who are attending family history clinics are being offered the opportunity to participate in a chemoprevention trial. Following genetic counselling about breast cancer risk and the nature of the trial, consenting women are randomised to either tamoxifen or a placebo for 5 years. We report a study comparing anxiety in 99 women participating in the tamoxifen prevention trial with that found in 87 women *with* a family history who are not attending a specialist clinic and 86 women *without* a reported family history. The non-trial women were all recruited from the National Breast Screening Programme (NBSP). Consequently, all the women in the study have undergone the same potentially anxiety-provoking experience of mammography.

Correspondence: K Thirlaway
Received 1 August 1995; revised 18 December 1995; accepted 19 December 1995

### Method

### *Subjects*

*Tamoxifen trial participants* A total of 550 women are currently participating in the psychosocial arm of the chemoprevention trial of tamoxifen in women at high genetic risk of developing breast cancer. In the psychosocial study the impact that long-term tamoxifen has on quality of life variables is being assessed. One hundred of the trial participants recruited from the Royal Marsden Hospital family history clinic were randomly selected for the comparative study being reported here.

Women were referred to the Royal Marsden by their general practitioners and had therefore received some information about risk before their visit. At the clinic they underwent clinical examination and mammography, followed by genetic counselling. Eligible women with a greater than 4-fold risk were told about the chemoprevention trial. All this information was repeated on a detailed information sheet that they were required to read before giving informed consent to joining the prevention trial.

*National Breast Screening Programme participants* A total of 131 women with a family history of breast cancer and 126 women without such a history were recruited from women attending for routine screening as part of the National Breast Screening Programme in south-east London. These women received no genetic counselling before recruitment. The women in all groups were aged 50 years or more.

Women were classified using the Registrar General Classification System according to their occupation: I, professional; II, semi professional; III, skilled; IV, unskilled and V, not classifiable. The final class V included housewives, students, voluntary counsellors and others with an occupation that did not fit the first four categories. Women participating in the prevention trial were more likely to be from groups I and II than women from the National Breast Screening Programme (chi-square test = 19.9, $P<0.0001$), but as the majority of women were unclassifiable by occupation (V) these data are an unreliable means of establishing the social class of the groups.

### *Assessment measures*

All subjects were given the Spielberger trait anxiety inventory, a standardised clinical tool for measuring anxiety (Spielberger *et al.*, 1983), and a questionnaire examining women's understanding of: perception of risk in the general population; causality of breast lumps; the relationship between age and breast cancer risk; and other demographic and behavioural variables associated with breast cancer risk (Fallowfield *et al.*, 1990). Women were asked to indicate whether they believed the general population risk to be 1 in 100, 1 in 55 or 1 in 12. These questionnaires had been used in a previously reported study of breast cancer screening (Fallowfield *et al.*, 1990).

Trait anxiety was used as a measure of anxiety for two reasons. Firstly, having an increased risk of developing breast cancer is a permanent condition so any negative effect on anxiety would be expected to be consistent and long-term. Secondly, as women in the study returned their questionnaires by post, ensuring they completed them at the same time was impossible, rendering a measure of state anxiety (how you feel right now) inappropriate.

### *Procedure*

Questionnaires were given to all women who consented to join the psychosocial arm of the tamoxifen prevention trial, at trial entry after mammography. They had been informed that the purpose of the psychosocial study was to assess the effect of tamoxifen on anxiety and other quality of life variables. Prepaid envelopes were provided for the return of the questionnaires.

Women at the national breast screening clinic were recruited into the study following their mammogram. They were given both questionnaires and asked to return them in prepaid envelopes. They were informed that the study aimed to monitor the impact of the screening programme on anxiety. Family history status was confirmed by the radiologist who routinely asked all women a number of sociodemographic and clinical questions including family history.

The entry criteria for women aged 50 or more to join the tamoxifen prevention trial were a mother or sister who has had breast cancer or two close relatives who have had breast cancer. The entry criterion for participation in the psychological study for women attending for routine screening mammography was at least one close maternal relative with breast cancer. Radiologists at the screening clinic did not have time to research family history in great detail. Therefore, it is possible that some women with a family history recruited from the NBSP would not have had sufficiently high risks to make them eligible for the tamoxifen prevention trial.

Trial participants and women from the screening programme were all given the questionnaires to take home and complete on the same day that they had their mammogram so time between mammography and completion of the questionnaires was similar for both.

### *Analysis*

The data were analysed using the SPSS/PC statistical package. Initially, a stepwise regression analysis was carried out to identify any variables related to anxiety. The potential variables entered into the regression analysis were: overall knowledge score, understanding of general population risk for breast cancer, understanding of hereditary nature of breast cancer, occupation, age, clinic attended, family history status.

The Mantel–Haenszel test for linear association was used to examine the relationship between occupation, recruitment centre and the two relevant factors generated from the regression analysis. A two-way analysis of variance: experimental group (tamoxifen trial participants/NBSP *with* history/NBSP *without* history) × factors generated from the regression analysis, was used to analyse anxiety scores. One-way analysis of variance was used to examine any significant interactions further. This is a conservative process that biases against any significant findings. *t*-Tests were used to compare group means.

### Results

### *Compliance*

*Tamoxifen trial participants* A total of 88.2% of women participating in the psychosocial arm of the tamoxifen trial returned their questionnaires. One hundred of these were randomly selected for the comparative study reported here. One woman had failed to complete the questionnaires so data from 99 women were available for analysis.

*National Breast Screening Programme participants* Questionnaires were given to 131 women with and 126 without a family history. A total of 105 (80%) of those with a history and 96 (76%) of those without a history returned the questionnaires. Owing to failure to complete the questionnaires correctly, returning them unanswered or only completing one questionnaire, data from 87 women *with* a family history and 86 *without* such a history were available for analysis.

### *Factors associated with anxiety*

Regression analysis showed that two variables, understanding of breast cancer risk in the general population and understanding of the hereditary nature of breast cancer, explained 3% (adjusted $R^2=0.03$) of the total variance in anxiety ($F=5.38$, $P=0.005$).

*The general population risk of developing breast cancer* In all, 47 (47.5%) of the tamoxifen trial participants recognised the general population risk of developing breast cancer to be 1 in 12. Significantly fewer women from the screening programme were accurate: 24 (27.6%) NBSP women *with* a history and 20 (23.3%) NBSP women *without* a history recognised that all women have a 1 in 12 chance of developing breast cancer in their lifetime (chi-square test = 8.07, $P=0.004$). In total there were 91 (33.5%) women aware of the general population risk.

**Table I** Anxiety scores in relation to awareness of population risk of breast cancer in women participating in the tamoxifen prevention trial compared with women with and without a family history of breast cancer undergoing routine screening

| | *Tamoxifen trial participants* | *Screening women with family history* | *Screening women without family history* |
|---|---|---|---|
| *Number of assessable women* | 99 | 87 | 86 |
| General population risk | | | |
| Aware | | | |
| Mean anxiety score | 37.5 (s.d. 9.8) | 45.5 (s.d. 9.5) | 39.3 (s.d. 10.2) |
| Number | 47 | 24 | 20 |
| Unaware | | | |
| Mean anxiety score | 36.8 (s.d. 8.8) | 38.3 (s.d. 10.9) | 39.5 (s.d. 9.8) |
| Number | 52 | 63 | 66 |

There was an interaction effect on anxiety between understanding of general population risk and the three experimental groups (trial participants, NBSP women *with* a history and NBSP women *without* a history) ($F=3.17$, $P=0.01$). That is to say only in the 91 women who were aware of general population risk did anxiety scores vary significantly in the three groups ($F=5.43$, $P=0.006$). Among these 91 accurate women, NBSP women *with* a family history had significantly higher anxiety scores than women participating in the tamoxifen trial ($t=3.35$, $P=0.002$) or NBSP women *without* a family history ($t=2.08$, $P=0.04$). Women participating in the tamoxifen trial had similar anxiety scores to NBSP women *without* a family history (Table I).

*Understanding of the herditary nature of breast cancer* Two-way analysis of variance revealed no relationship between understanding of the hereditary nature of breast cancer and experimental group on anxiety.

### Discussion

Understanding of general population risk only accounted for 3% of the variance in anxiety found in the entire sample. This was not unexpected as the relationship found between risk perception and anxiety in this study was related to both 'risk status' and 'clinic attended'. Therefore the relationship existed only in a small group of the entire study population and did not account for a large percentage of total variance.

A total of 91 (33.5%) of the entire sample of 272 women correctly understood the general population risk of developing breast cancer to be 1 in 12 (Table I). Almost half, 47 of 99 (47.5%) of the women participating in the tamoxifen trial were aware of this figure in comparison with 45 of 173 (26%) of the NBSP women with or without a family history. This suggests that the genetic counselling offered at the family history clinic did improve understanding and is compatible with other studies of population risk perceptions in 'at risk' women attending a family history clinic. (Evans *et al.*, 1993). Fallowfield *et al.* (1990) found that only 14% of women attending for routine screening at the same NBSP centre had realistic risk perceptions. It would seem that there has been some improvement in understanding of breast cancer risk in women attending for routine screening during the past 5 years.

Amongst the 91 accurate women, the 24 *with* a family history not attending a specialist clinic had significantly higher anxiety scores than the 47 with a history participating in the tamoxifen trial (Table I). This is despite the less stringent criteria of family history used as a definition of 'high risk' for women from the NBSP. These data do not add support to the hypothesis that specialist clinics see a self-selected group of the most anxious. Women participating in the tamoxifen trial had similar anxiety scores to women *without* a family history attending for routine screening. This was true for women with accurate or inaccurate risk perceptions.

One explanation of the findings is that women with accurate risk perceptions who are highly anxious avoid specialist clinics. Alternatively, participation in the prevention programme or attendance at a specialist clinic may alleviate anxiety in 'high risk' women with accurate risk perceptions. Kash *et al.* (1992) and Lerman and Croyle, (1994) reported high levels of anxiety in American women attending a breast cancer family history clinic *but not* participating in any prevention programme which suggests it may be participation in the prevention programme rather than attendance at a clinic which is related to any alleviation of anxiety. However, there are no data from American 'at risk' women *not* attending any specialist clinic; they too may have higher levels of anxiety than those reported in American woman attending a history clinic.

The data reported in our study suggest that participation in the tamoxifen prevention programme, which requires regular screening and attendance at a family history clinic does not exacerbate anxiety and may even alleviate anxiety for some women.

**Acknowledgements**
The authors wish to acknowledge the help of staff running the tamoxifen prevention trial at the Royal Marsden Hospital and staff and radiographers at the Breast Screening Centre, Camberwell Green. We also thank Dr Jack Cuzick for statistical advice and the CRC for their financial support.

### References

EVANS DGR, BURNELL LD, HOPWOOD P AND HOWELL A. (1993). Perceptions of risk in women with a family history of breast cancer. *Br. J. Cancer*, **67**, 612–614.

FALLOWFIELD LJ, RODWAY A AND BAUM M. (1990). What are the psychological factors influencing attendance, non-attendance and re-attendance at a breast screening centre? *J. R. Soc. Med.*, **83**, 547–551.

HOWE HL. (1981). Social factors associated with breast self-examination among high risk women. *Am. J. Pub. Health*, **71**, 251–255.

KASH KM, WEINBERG GB, SMALL A AND HENDON MS. (1992). Breast cancer screening among relatives of women with breast cancer. *Am. J. Pub. Health*, **81**, 1174–1179.

LERMAN C AND CROYLE R. (1994). Psychological issues in genetic screening for breast cancer susceptibility. *Arch. Int. Med.*, **154**, 609–616.

SPIELBERGER C. (1983). *Manual for the State-trait Anxiety Inventory*. Consulting Psychologists Press: Palo Alto, CA.